# Giant Lipoma‐Like Liposarcoma of the Mediastinum: A Rare Case of Acute Respiratory Failure

**DOI:** 10.1002/ccr3.70495

**Published:** 2025-05-09

**Authors:** Lorenzo Giovannico, Gerardo Cazzato, Andrea Marzullo, Tomaso Bottio

**Affiliations:** ^1^ Cardiac Surgery Unit, Department of Precision and Regenerative Medicine and Ionian Area (DiMePRe‐J) University of Bari Aldo Moro Bari Italy; ^2^ Section of Molecular Pathology, Department of Precision and Regenerative Medicine and Ionian Area (DiMePRe‐J) University of Bari Aldo Moro Bari Italy

**Keywords:** acute respiratory failure, clamshell incision, histopathological diagnosis, lipoma‐like variant, mediastinal liposarcoma, surgical resection

## Abstract

This report presents a rare case of a 60‐year‐old man with acute respiratory failure due to a giant lipoma‐like liposarcoma. Following the successful surgical removal of the 2850‐g mass, the patient's respiratory function improved immediately. This case highlights the importance of diagnosis and surgical intervention in managing large mediastinal tumors.

## 1 Introduction

Liposarcomas are rare malignant tumors of adipocytic origin, with mediastinal localization being extremely uncommon. Among its subtypes, the lipoma‐like variant is particularly rare and can mimic benign lipomas, making early diagnosis challenging. We present a case of a giant mediastinal lipoma‐like liposarcoma causing acute respiratory distress, requiring immediate surgical intervention.

## 2 Case Report

A 60‐year‐old man suddenly experienced respiratory distress, leading to an urgent transfer to the emergency department. The patient presented with marked hypoxia and was quickly intubated. An AngioCT scan of the thorax and abdomen was performed.

**FIGURE 1:**
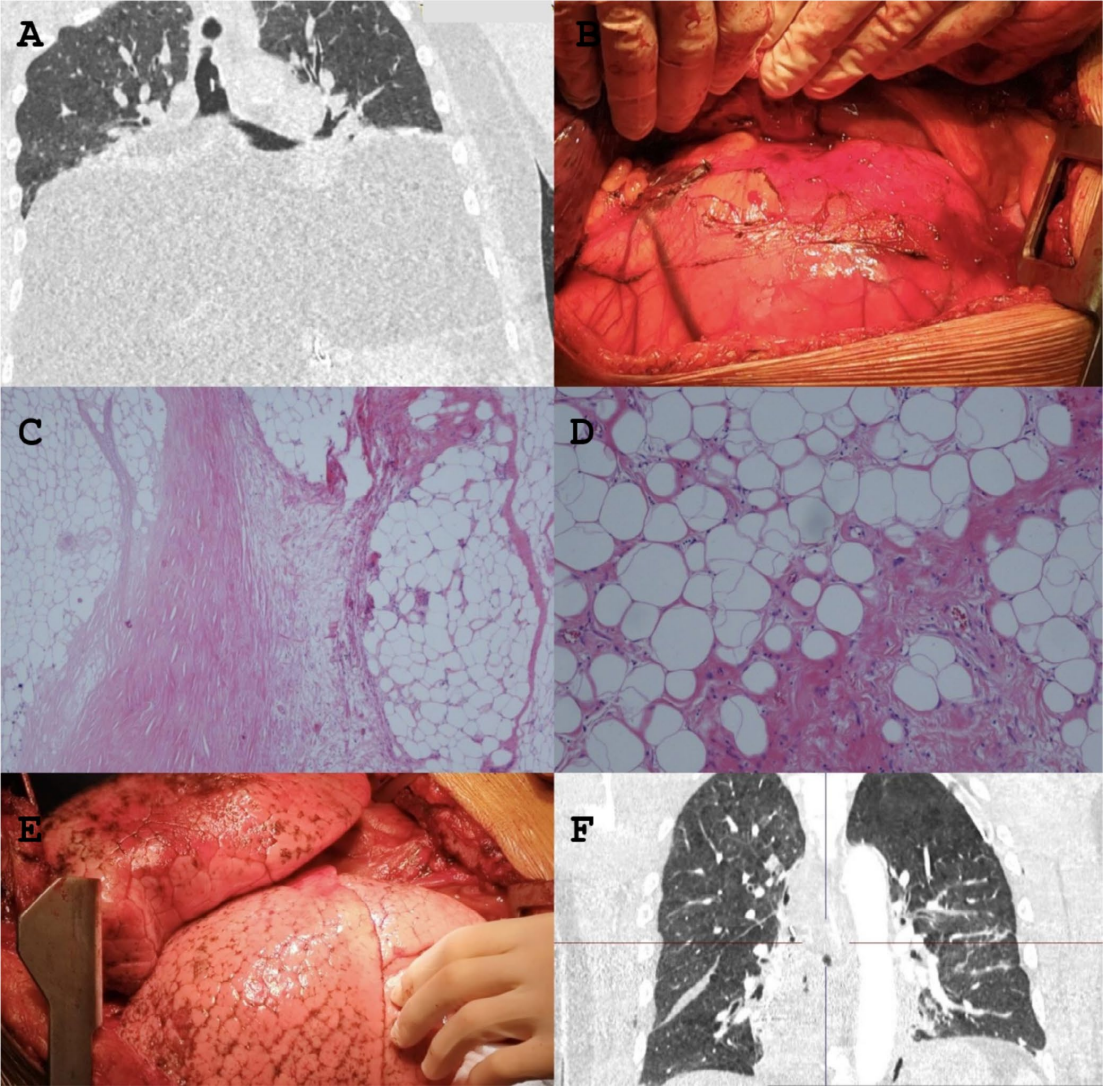
(A) CT scan show a gigantic thoracic mass that completely occupies the posterior mediastinum; (B) The intraoperative image shows, once the lung has been lifted and moved, the thoracic mass documented on the CT examination; (C) Histological microphotograph showing an area of lipoma‐like liposarcoma (on the right) separated by a sclerotic band from a necrotic area (on the left) Hematoxylin–Eosin 40× original magnification; (D) Atypical cells with asymmetrical shape of the neoplastic adipocytes; rare lymphocytes are scattered in the stroma. Hematoxylin–Eosin 100× original magnification; (E) The intraoperative image shows, once the mass has been completely resected, the re‐expansion of the lungs; (F) The postoperative CT scan shows complete expansion of the lungs and complete resection of the mediastinal liposarcoma mass.

The CT scan revealed a gigantic thoracic mass (Figure 1A,B). The patient underwent surgery via clamshell access, and the mass was completely excised. The total weight of the mass was approximately 2850 g.

Histological examination confirmed the presence of a lipoma‐like liposarcoma with areas of necrosis (Figure 1C). Hematoxylin–Eosin staining at 40× magnification highlighted atypical adipocytic cells separated by a sclerotic band. Scattered lymphocytes were observed in the stroma (Figure 1D).

Postoperatively, the patient showed immediate respiratory improvement and was extubated the following morning. A follow‐up CT scan confirmed complete lung re‐expansion (Figure 1E,F).

## 3 Discussion

Primary mediastinal liposarcomas are exceedingly rare and often asymptomatic until they reach significant sizes. This case is particularly notable due to the acute presentation with respiratory failure, necessitating urgent surgical intervention. Previous literature describes similar cases [1, 2, 3], but often with chronic respiratory symptoms rather than acute failure. The surgical approach via clamshell incision allowed complete resection, significantly improving prognosis. Histopathological confirmation is critical for differentiating between liposarcomas and benign lipomas, as the latter require less aggressive management.

## 4 Prognostic and Follow‐Up Considerations

While complete surgical excision is the standard treatment, liposarcomas carry a risk of local recurrence. Regular follow‐up imaging is essential, with MRI or CT scans every 6 months for at least 3 years. The patient will undergo periodic surveillance to monitor for potential recurrence.

## Author Contributions

**Lorenzo Giovannico:** conceptualization, writing – original draft, writing – review and editing. **Gerardo Cazzato:** methodology, writing – original draft. **Andrea Marzullo:** conceptualization, supervision, writing – review and editing. **Tomaso Bottio:** data curation, formal analysis, supervision, writing – review and editing.

## Consent

The authors should confirm that written informed consent has been obtained from the involved patient. Written informed consent was obtained from the patient to publish this report in accordance with the journal's patient consent policy.

## Conflicts of Interest

The authors declare no conflicts of interest.

## Data Availability

Data sharing is not applicable to this article as no datasets were generated or analyzed during the current study.
